# Spatiotemporal Dynamics of Hantavirus Cardiopulmonary Syndrome Transmission Risk in Brazil

**DOI:** 10.3390/v11111008

**Published:** 2019-10-31

**Authors:** Renata L. Muylaert, Gilberto Sabino-Santos, Paula R. Prist, Júlia E. F. Oshima, Bernardo Brandão Niebuhr, Thadeu Sobral-Souza, Stefan Vilges de Oliveira, Ricardo Siqueira Bovendorp, Jonathan C. Marshall, David T. S. Hayman, Milton Cezar Ribeiro

**Affiliations:** 1 Department of Ecology, Institute of Biosciences, São Paulo State University (UNESP), Rio Claro 13506-900, Brazil; juemioshima@gmail.com (J.E.F.O.); bernardo_brandaum@yahoo.com.br (B.B.N.); miltinho.astronauta@gmail.com (M.C.R.); 2 Molecular Epidemiology and Public Health Laboratory, Hopkirk Research Institute, Massey University, Private Bag 11-222, Palmerston North 4474, New Zealand; J.C.marshall@massey.ac.nz (J.C.M.); D.T.S.Hayman@massey.ac.nz (D.T.S.H.); 3 Center for Virology Research, Ribeirão Preto Medical School, University of São Paulo, Av. Bandeirantes 3900, Vila Monte Alegre, Ribeirão Preto 14049-900, Brazil; sabinogsj@gmail.com; 4 Vitalant Research Institute, 270 Masonic Avenue, San Francisco, CA 94118, USA; 5 Department of Laboratory Medicine, University of California, San Francisco, 270 Masonic Avenue, San Francisco, CA 94118, USA; 6 Department of Ecology, Biosciences Institute, University of São Paulo, São Paulo 05508-900, Brazil; pprist@hotmail.com; 7 Centro Nacional de Pesquisa e Conservação de Mamíferos, Carnívoros (CENAP), Instituto Chico Mendes de Conservação (ICMBio), Estrada Municipal Hisaichi Takebayashi, 8600—Bairro da Usina, Atibaia 12952-011, Brazil; 8 Instituto Pró-Carnívoros, Av. Horácio Neto 1030, Parque Edmundo Zanoni Atibaia 12945-010, Brazil; 9 Department of Botany and Ecology, Federal University of Mato Grosso (UFMT), Cuiabá 78060-900, Brazil; thadeusobral@gmail.com; 10 Departamento de Saúde Coletiva da Faculdade de Medicina, Universidade Federal de Uberlândia, Avenida Pará, 1720, Campus Umuarama, Uberlândia 38405-320, Brazil; stefanbio@yahoo.com.br; 11 Departamento de Ciências Biológicas, Universidade Estadual de Santa Cruz, Ilhéus 45662-900, Brazil; siqueira.sal@gmail.com

**Keywords:** land use change, emerging diseases, public health, polygon-based analysis, approximate Bayesian inference, latent Gaussian models, integrated nested Laplace approximations, zero inflation

## Abstract

Background: Hantavirus disease in humans is rare but frequently lethal in the Neotropics. Several abundant and widely distributed Sigmodontinae rodents are the primary hosts of *Orthohantavirus* and, in combination with other factors, these rodents can shape hantavirus disease. Here, we assessed the influence of host diversity, climate, social vulnerability and land use change on the risk of hantavirus disease in Brazil over 24 years. Methods: Landscape variables (native forest, forestry, sugarcane, maize and pasture), climate (temperature and precipitation), and host biodiversity (derived through niche models) were used in spatiotemporal models, using the 5570 Brazilian municipalities as units of analysis. Results: Amounts of native forest and sugarcane, combined with temperature, were the most important factors influencing the increase of disease risk. Population at risk (rural workers) and rodent host diversity also had a positive effect on disease risk. Conclusions: Land use change—especially the conversion of native areas to sugarcane fields—can have a significant impact on hantavirus disease risk, likely by promoting the interaction between the people and the infected rodents. Our results demonstrate the importance of understanding the interactions between landscape change, rodent diversity, and hantavirus disease incidence, and suggest that land use policy should consider disease risk. Meanwhile, our risk map can be used to help allocate preventive measures to avoid disease.

## 1. Introduction

Land use change can influence the spread and distribution of infectious diseases, especially emerging zoonotic ones [1], such as hantavirus disease, caused by a nearly globally distributed set of *Orthohantavirus* genotypes (hereafter hantavirus) [2] (family *Hantaviridae* [3,4]). In Brazil, hantavirus disease is manifested in humans as a cardiopulmonary syndrome (HCPS) [5], and it is considered a problem due to its high mortality rates [6]. The risk factors for human hantavirus infection include direct exposure to rodent hosts’ blood, saliva and excreta [7], which is a consequence of contact during agricultural or leisure activities [8,9].

There is evidence that agriculture expansion—particularly of sugarcane plantations— influences hantavirus disease risk in southeastern Brazil [10]. Sugarcane, planted pasture, and *Eucalyptus* plantations may increase HCPS outbreaks [11]. Maize plantations were linked to hantavirus disease in previous works [12,13]. Human occupied areas in proximity to native vegetation, however, also tend to present a higher disease risk because they benefit rodent hosts and allow higher probability of local human encroachment and contact [14,15].

There were notable changes in Brazilian land use from 1940 until recently [13]. Besides deforestation, there was an increase in sugarcane and maize plantations, expansion of soybean areas, and intensification in the use of pre-existing pasture areas. These changes may have expanded the distribution of rodent host species, as several of them can utilize multiple habitats [16], and consequently increased human exposure to hantaviruses.

Hantavirus disease in different localities is apparently caused by only one virus that infects people in each region and the virus is typically linked to a main opportunistic host species. If there is only one competent host species, greater species richness possibly dilutes transmission to humans by increasing non-competent host contacts. In this one-species, one-host situation, the dilution effect will reduce the chance of transmission through non-competent host contacts. Therefore, the dilutive effect on the predominant viral lineage increases with the abundance and number of other hosts to reduce the chance of viral transmission. Contrastingly, if multiple competent hosts exist, increased diversity may increase risk. The virus-host species specificity paradigm does not seem to be completely true for hantaviruses in South America [17]. There are at least three *Orthohantavirus* species known to have the potential to cause disease in humans in Brazil (*Andes orthohantavirus*—ANDV, *Laguna Negra orthohantavirus*—LANV, and *Rio Mamoré orthohantavirus*—RIOMV, a LANV variant) and they can be detected in more than 20 rodent species in South America, with considerable range overlap between some of them [17]. Therefore, it is necessary to investigate several rodent species beyond the known individual reservoir host species [10,18]. Mapping the areas where hantavirus host species are distributed and may concentrate different hantavirus genotypes is a priority for disease surveillance [9]. There have been recent attempts to understand HCPS distribution regionally [19] and nationally [12], including using expert opinion approaches to develop and analyze vulnerability measures [6,20]. However, to our knowledge, investigations modeling the disease risk for large regions are still missing in the Neotropics, particularly for Brazil. These studies are important to help understand the drivers increasing hantavirus risk and to guide educational and preventive measures.

The transmission dynamics of hantaviruses to humans and disease surveillance is a complex process, especially in the hyper-biodiverse South America [21], for which case notifications depend on several steps. The rodent host needs to occur in a certain location, and, for the infection to exist, rodent populations may need to be above a certain abundance threshold [11]. Then excreted virus particles in substrates (e.g., soil) must be aerosolized under specific environmental conditions, infecting humans through inhalation of viral particles. Thereafter, susceptible humans must develop disease and present themselves to clinics with clinical signs and symptoms that must be diagnosed as hantavirus, prior to a compulsory report being provided to the Ministry of Health.

We believe that areas with higher numbers of at-risk people (adults working in rural areas) in the State of São Paulo [6] have higher disease risk due to the increased probability of exposure to pathogens. We expect that increased proportions of rural workers increases hantavirus disease risk. We also expect that areas of moderate to high levels of rainfall have increased hantavirus disease incidence as an indirect effect on host species population growth (an increase in density—bottom up regulation [11]). Rodent populations benefit from mild temperatures [22] and virus viability in the environment seems to be higher in mild temperatures (virus is inactivated after 24 h in temperatures ≥ 37 °C) [23].

Here, we aimed to (1) generate disease risk maps using the presence and the number of notified hantavirus disease cases over a 24 year data series as response variables, and the following hypothesized risk factors as predictor variables (Figure S1): social vulnerability, climate, land use change, and biodiversity; and (2) evaluate the ability of the best supported model to predict new confirmed cases in humans, using subsets of our data series. Our expectations were that land use change—especially the agricultural expansion of sugarcane and maize—and social vulnerability are the main predictors of disease risk. We also expect that host diversity positively influences the probability of disease cases in humans. This is the first study, to our knowledge, that considers all these components together. We consider that this is an important step to be taken to investigate HCPS dynamics, since the spatiotemporal analysis can elucidate how multiple factors modulate the distribution of the disease.

## 2. Materials and Methods

### 2.1. Hantavirus Disease Cases and Population Data

Data on human notified cases from 1993 to 2016 were provided by the Ministry of Health (http://www2.datasus.gov.br/DATASUS/index.php?area=0203&id=29878153). From 1993–1999, all cases were reported as notified (not necessarily laboratory confirmed). After 2000, notified and laboratory confirmed cases were available, and we used this second set of data here. A zero inflated modelling approach was used using two response variables: (1) the presence and (2) the counts of positive cases in a municipality in one year, using the data from Brazilian Ministry of Health for the period. Geographical precision of cases is reported at the municipality level because of the privacy policy for infected people. We used as the spatial reference the polygons of 5570 Brazilian municipalities taken from 2015 (after removing two lakes) from the Instituto Brasileiro de Geografia e Estatística available at (ftp://geoftp.ibge.gov.br/organizacao_do_territorio/malhas_territoriais/malhas_municipais/municipio_2015/Brasil/BR/).

All covariates were aggregated to the municipality level. We extracted data directly from Brazilian government databases (rural workers data) or applied zonal statistics for other data sources (Table S1), using the average or amount (%) for the environmental predictors and counts of species for the biodiversity component The selected indicator for social vulnerability was “population at risk”, which was the number of rural workers 18 years old or older. Population censuses were conducted at three time periods (1991, 2000, and 2010). Since we had three censuses per municipality in the study time period, we used the function approxExtrap to estimate population growth by using linear interpolation of the number of rural workers. All covariates were centered to zero, by subtracting the mean from each value and dividing by the standard deviation.

### 2.2. Potential Host Richness

We define hosts as the rodent species which may contribute to the maintenance of hantavirus infection. We selected the following species after evaluating the availability of occurrence data for modeling: *Akodon cursor*, *Akodon montensis*, *Calomys tener*, *Holochilus sciureus*, *Necromys lasiurus*, *Oligoryzomys eliurus* [24], *Oligoryzomys fornesi*, *Oligoryzomys microtis*, *Oligoryzomys nigripes*, and *Oxymycterus dasytrichus*. We used as predictors the count of unique host species (host richness) per municipality.

We spatially predicted the occurrence of the main hantavirus hosts in Brazil using Ecological Niche Models (ENMs) [25]. The list of target species used the most recent reviews on rodent host data [17,21]. Rodent species occurrence data was mined from PREDICT (https://www.usaid.gov/news-information/fact-sheets/emerging-pandemic-threats-program), Species Link (http://www.splink.org.br/), Vert Net (http://vertnet.org/), Cerrado small mammals [26], and Atlantic small mammals [27]. Data mining was finished on August 10, 2018. We filtered the number of records to one per cell (1/km^2^) for each species. We calculated habitat suitability for each rodent species that we managed to have enough records (*n* = 10) after applying a geographical filtering. The modelling spatial extent was defined as the region that includes all Brazilian biomes: Amazônia, Cerrado, Atlantic Forest, Caatinga, Pampa, and Pantanal. We added a buffer of 220 km around the species occurrence points found for each species to avoid problems on the border of predictions [28]. Climate predictors were downloaded as raster files delimited by the area of each species occurrence plus a 220 km buffer. Climate predictors for the ENM were selected with a factorial analysis of Worldclim 2.0 climatic variables (http://www.worldclim.org/bioclim) over the extent regions (~1 km spatial resolution). We built ENMs using four algorithms [29]: SVM [30], Bioclim [31], MaxEnt [32], and Gower distance [33], since ensemble forecasting approaches produce stronger predictions and are considered a useful framework to account for uncertainties in model projections [12]. Resampling of data points was conducted with bootstrapping with a convergent threshold of 10^−5^ considering 10,000 background points. To evaluate model performance, we randomized the occurrence data into 75%:25% train:test samples to calculate the True Skill Statistics (TSS) and the Area Under the Operator Curve (AUC) [34] for each model. We used expert opinion for selecting the most realistic maps, using the weighted suitability based on the best-supported models (TSS > 0.5).

Threshold values were calculated to transform each of the model predictions (probabilities, distances, or similar values) to a binary score (presence or absence of each species). We generated the maps based on maximum specificity and sensitivity thresholds at which the sum of the sensitivity (true positive rate) and specificity (true negative rate) was highest. After selecting the best supported ENMs we plotted the binary maps based on (1) the lowest presence threshold (LPT) of observed presences and (2) on the 10th percentile of the LPTs, and asked for expert opinion. After selecting the best maps for each species, we calculated the sum of presences of each host per pixel summing the binary maps. The overlay of species layers resulted in a final map of host–species richness. Then, we derived zonal statistics based on the host species map for the Brazilian municipalities’ shapefile, calculating the predicted host richness per municipality.

### 2.3. Land Use and Climate

For calculating the amount (%) of pasture and grasslands we used the compiled Brazilian Historical Agricultural Land Use database [13] (see data sources at Table S1), which covers most of the time series (1993–2014). From this dataset we extracted sugarcane, maize, planted pasture, and native pasture land-use classes per municipality per year. Forest and forestry amount per municipality were calculated with Mapbiomas collection 2 for 2000–2016 (www.mapbiomas.org). We made a mosaic raster from Mapbiomas 2 using the codes 1 to 8 as “native forests”, which produced binary maps of forests from 2000 to 2016. We did the same for forestry (mostly *Eucalyptus* and *Pinus* plantations) using the code 9. Then, we extracted the amount (%) of each of those land use classes in each municipality. Rainfall data was extracted from Climate Hazards Group Infrared Precipitation with Stations (CHIRPS) [35], and surface temperature data from NOAA Merged Land Ocean Global Surface Temperature Analysis Dataset [36]. We calculated average annual rainfall (mm) from monthly data, and for temperature we used average annual temperature (°C*10). Zonal statistics for municipalities were calculated from custom functions available at https://github.com/LEEClab/GeneralizedZonalStats. We used the WGS 84 coordinate reference system throughout and kept the original spatial resolution of data (Table S1).

### 2.4. Spatiotemporal Models

We evaluated the distribution of hantavirus disease cases across space and time to select the most appropriate likelihood distributions used in the models. We did that by calculating the proportion of zeros in the dataset. To our knowledge, transmission between humans has not been reported for hantavirus genotypes from Brazil [37]. The absence of human-to-human transmission helps keep the disease rare. Modeling a rare and emergent disease is challenging, but given the size of the dataset and number of cases (“successes”), it is possible to fit comprehensive models to it. For instance, Prist et al. [10] had 1.6% “events” in São Paulo State and it was possible to infer risk with considerable accuracy. We decided to use data from 2000 onwards, from where the consistency of case presences (more than 1%) allowed us to model the events. The presence of cases in a municipality from 2000–2014 (i.e., 15 years) in 5570 municipalities is equivalent to 1.1%, where a case was present 958 times over 83,550 observations. We used the “zeroinflatedpoisson0” distribution for the zero truncated model [38]. We used data from 2000–2014 to fit the model and data from 2015–2016 for checking the correspondence between predictions and observed data not included in the model.

We decided to use a zero inflated approach, considering two models: (1) a model containing a binomial distribution to estimate the probability of having any case in a municipality (risk), using presence or absence of cases as response variable; and (2) a zero-truncated Poisson model for the count data, where we only use as the input data observations in municipalities where the number of cases were different from 0 as response variable. The covariates used in both models were the same (see Figure S1). We verified the model sensitivity using the most recent covariate data and new case data, corresponding to 2015–2016. Prior to model building, we checked for correlation between model predictors and variance inflation factors (Figure S2). We ran models with the components based on Figure S1. We set model parameters with non-informative priors for all covariates.

Municipalities or political limits could be used in our analyses, but careful interpretation of model predictions should be made because of the bias of using municipality limits at data boundaries. A stratified spatial autocorrelation Moran’s I for each year was applied for data exploration (Table S2). Then, we created a default neighbor list object and graph from the polygon’s shapefile containing the spatial interaction among the municipalities. We used a second-order random walk as the temporal term (rw2) and a Besag iCAR spatial model, which is able to cope with irregularly spaced data [39]. To reduce model complexity and avoid computational limitations, interaction terms were not included.

We inspected the model fit [39] using uncertainty calculations and the conditional predictive ordinate (CPO) values as a measure of model adequacy. CPO is a type of cross-validation procedure that ranges from 0 to 1 for each observation. If CPO values were inadequate, an optimization of the model was conducted to improve estimates using inla.cpo. The uncertainty in risk predictions was calculated based on the amount of variation of 95% highest and lowest credible intervals from the posterior mean. All model response variables used were related to data from 2000–2014. After diagnosing the spatiotemporal models (a model containing a Bernoulli component and a model containing a truncated Poisson component), we applied them to new data for covariates from 2015–2016. This allowed us to assess the model validation [40].

For the spatial analysis and raster processing we used Python 2.7 [41], GRASS GIS 7.2 [42] and QGIS 2.18 [43]. For ENMs’ development we used the package *dismo* [44] and for spatiotemporal model building we used the package INLA 19.01.24 [45,46] in R 3.5.1 [47]. Code developed for data analyses are available at a public repository *(*https://github.com/renatamuy/Muylaert_et_al_2019*).* Coefficient plots were created using coefINLA (https://github.com/hesscl/coefINLA).

## 3. Results

### 3.1. Hantavirus Disease Cases and Population Data

**Figure 1:**
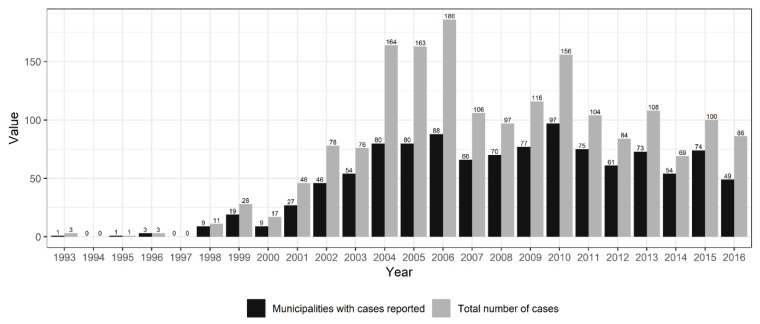
Hantavirus disease cases in Brazil. Data were downloaded from www.datasus.gov.br. Data from 2016 was updated in January 2019.

**Figure 2:**
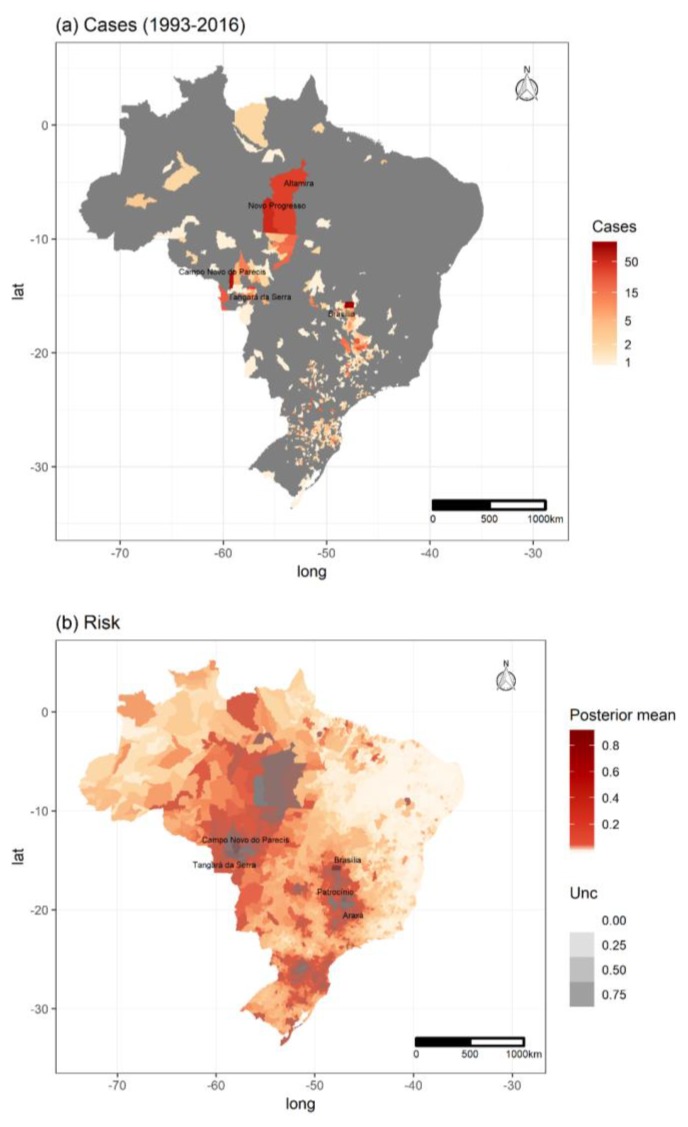
Hantavirus disease distribution in Brazil. (**a**) Observed values from 1993–2016, highlighting the municipalities with largest number of cases. Grey areas are municipalities with no notified cases; (**b**) Expected values for the probability of hantavirus disease in humans, predicted by a spatiotemporal model containing forest, climate, and social vulnerability, with uncertainty as transparency levels (Unc) based on the variation of credible intervals. The top five municipalities in terms of risk per year are highlighted. See the risk map without the uncertainty layer in Figure S4.

**Figure 3:**
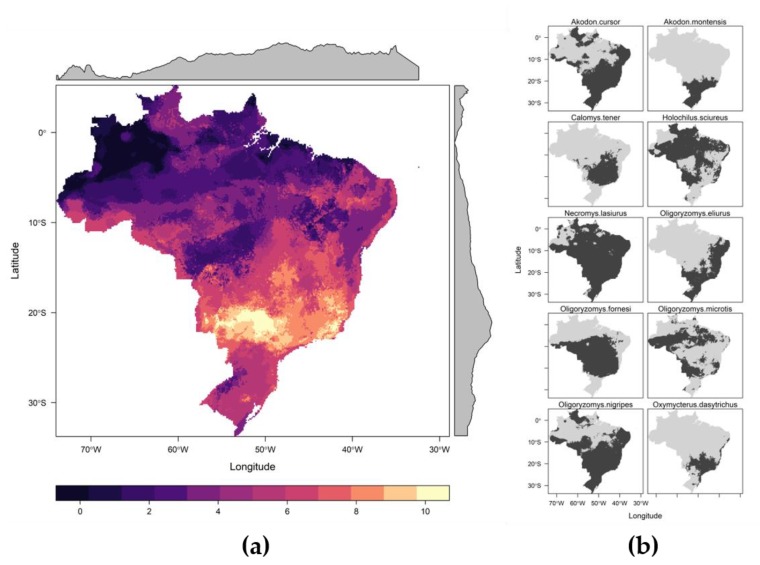
Distribution of potential hantavirus hosts in Brazil: (**a**) Richness of potential hantavirus hosts in Brazil. Lighter colors represent more hosts. The grey areas represent variation in pixel counts per latitude and longitude. (**b**) Binary maps generated by Ecological Niche models with the black areas indicating higher habitat suitability for each modeled rodent species.

**Figure 4:**
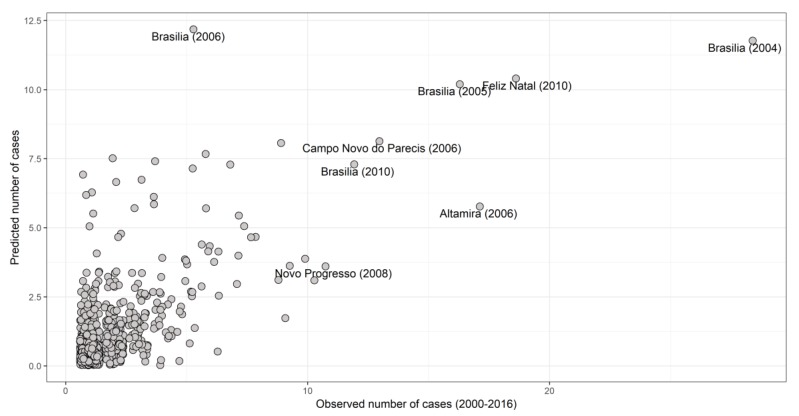
Scatterplot with the number of HCPS cases observed and predicted by a zero truncated Poisson model. Each point represents the number of hantavirus disease notified cases in a municipality in one year.

**Figure 5:**
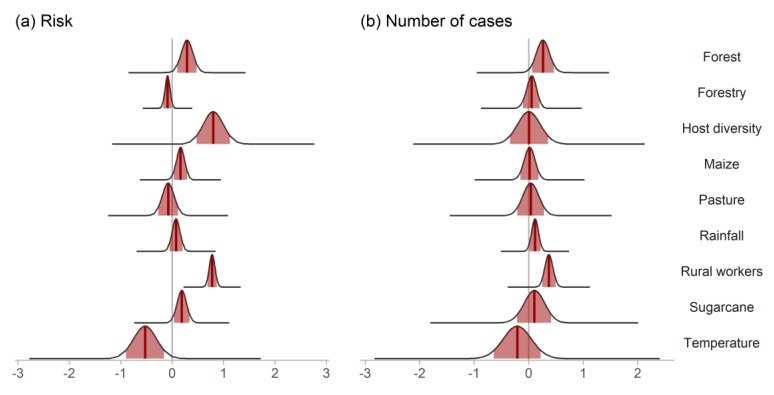
Posterior distributions of the effect sizes with the median (dark red line) and 95% credible intervals (light red shade) of each covariate on the (**a**) probability of cases and (**b**) positive counts of cases in a zero-truncated Poisson model, according to different predictors from two spatiotemporal models in Brazil from 2000–2014.

Overall, in the time period investigated, there was intense land use change in Brazil and also an increase in the number of HCPS cases (Figure 1 and Figure S3). Cases were reported in all major regions of the country, but most cases were reported in central and south Brazil (Figure 2a), where host richness values were also high (Figure 3). Population at risk (the number of rural workers) positively influenced both number of HCPS cases in a municipality and disease risk (Figure 4 and Figure 5).

Table S5 shows the average and range values for each fixed covariate. There was a clear increase of sugarcane, a decrease in number of rural workers through time (rural exodus), and a slight increase in forestry and decrease of pasture and maize amounts. The amount of forest also varied through time, as did temperature and rainfall. The least dominant culture was forestry, never reaching more than 2% on average, while pasture reached more than 20% on average, considering all municipalities in the country.

Among all predictors the variance inflation factor values were below 2 for the entire period and for the period selected for modelling (Figure S1). From the two models (Figure 4 and Figure 5), CPO values ranged from zero to 0.99, with a mean of 0.98 for the binomial model. CPO values for the truncated Poisson model ranged from zero to 0.96, with a mean of 0.36, indicating a “low” adjustment. Hence, we optimized the zero truncated Poisson model to improve the CPO values, resulting in a new mean of 0.58 (min = 0, max = 0.96).

### 3.2. Potential Host Richness

The following bioclimatic variables were used in the ecological niche models (Table S3): Mean Diurnal Range (bio02), Isothermality (bio3), mean temperature of warmest quarter (bio10), precipitation of driest quarter (bio17), and precipitation of the warmest quarter (bio18). We were able to run models for 10 species relevant for hantavirus infection [48]: two rodents of the genus *Akodon* (hosts of *Juquitiba orthohantavirus* and *Juquitiba-like orthohantavirus* [17], *Pergamino orthohantavirus* and *Jabora orthohantavirus* [48])—*Akodon cursor* and *Akodon montensis* [49]; one species of genus *Calomys* (hosts of *Laguna Negra orthohantavirus*, *Andes orthohantavirus* and *Andes-like orthohantavirus*, [17]): *Calomys tener*; one species of the genus *Holochilus* (*Rio Mearim orthohantavirus* in Brazil [50]), *Holochilus sciureus*; *Necromys lasiurus* (*Araraquara orthohantavirus*—ARQV, and *Juquitiba orthohantavirus* [17]); *Oligoryzomys eliurus* (part of the *Oligoryzomys* complex hosting ANDV) [24,51]; *Oligoryzomys fornesi* (*Anajatuba orthohanavirus* [24]); *Oligoryzomys microtis* (*Rio Mamore orthohantavirus* [24]), *Oligoryzomys nigripes* (*Juquitiba orthohantavirus* [17]); and *Oxymycterus dasytrichus* (ANDV clade, data not published [52,53]), for which antibodies against ARQV nucleoprotein were found, implying that this rodent can become infected. We did not have enough data to model *C. laucha* or *C. callosus*, the hosts of LANV, and they were excluded from the analysis. The summary of adequate ensemble models can be viewed in Table S4. Three expert researchers agreed on the 10 percentile maps as being the most accurate for all modelled species (Figure 3). Peaks of host richness values occur in central and southeastern Brazil (Figure 3). Host richness had a positive effect on disease probability (Figure 5a) but not on the number of cases (Figure 5b).

### 3.3. Land Use, Population and Climate

Increased maize, forest, and sugarcane amounts had a positive effect on disease probability. Temperature had a negative effect on case probability (Figure 5a). For the case counts, the predictors rainfall, forest, and number of rural workers had a positive effect, and no covariate presented a relevant negative effect (Figure 5b). When we evaluated all components, the effect of forest amount and rural workers consistently positively influenced both disease risk and number of cases in a municipality.

### 3.4. Spatiotemporal Models

There was spatiotemporal variation in HCPS risk during the period modelled (GIF S1). The coefficient values for each predictor in both models are in Table S6. Note that many municipalities in southeastern Brazil were not at risk (clear areas in Figure 2b and Figure S4), while some municipalities in the northeast with no cases reported were indicated as areas at risk. Figure 5 shows the effect of each covariate on the case probability (disease risk) and number of cases. Overall, four spatial higher risk conglomerates can be recognized, being two in the northwest, one in central southeastern areas and one in the south (Figure 2b).

The mean estimated probability parameter for zero (absence of HCPS cases) was estimated as $\pi_{zero}=0.99$, which is very high [39], but not surprising considering hantavirus disease rarity [11]. The time trend for the binomial model had a precision for year τ = 34.38 (*sd* = 29.71) (Figure S4a). For the temporal term random effect in the case count model (Figure S5a), the predicted effect on the number of cases seems to decrease with time (Precision for year τ=179.33, sd= 431.74). The estimated time trend predicted when using new data for the covariates from 2015 to 2016 did not differ from the overall trend (Figure S5b and Figure S6b). There was a non-linear temporal trend, with a slight increase in temporal correlation along the time series. Regarding spatial effects, the neighbor list object with 5570 regions had 32,546 non-zero spatial links, and 5.84 average number of links per municipality. The spatial random fields for the binomial model are shown in Figure S7 ($mean\;precision\;for\;u_{i}=0.19$, $\sigma_{i}=0.017$), and for the case counts, ($mean\;precision\;for\;u_{i}=0.5$), and $\sigma_{i}=0.09$. The predictions of the zero truncated model had a Pearson’s correlation 0.48 with the observed number of cases when we considered the period from 2000–2016, omitting the data from 2016 in the model (Figure 4). The higher risk biomes in terms of disease risk were Cerrado, Amazônia, and Atlantic Forest (Figure S8).

We provide the list of municipalities and their overall risk based on our model predictions in Table S7. Based on the observed number of cases, the largest outbreaks occurred in Brasília (2004, *n* = 28), Feliz Natal (2010, *n* = 19), and Altamira (2006, *n* = 17). The mean risk considering the entire country in the period modeled (2000–2014) was low (mean = 0.01) and the maximum risk reached 0.92 in Brasília in 2010. Considering a threshold of 5% for a relevant risk per municipality averaged over period modelled, 11.59% municipalities would be at risk. A threshold of 10% would include only 306 municipalities (5.4%). A high threshold for a risk value (>50%) would include only 11 municipalities, which (with the short State name and biome in parenthesis) are: Brasília (DF, Cerrado), Campo Novo dos Parecis (MT, Cerrado), Patrocínio (MG, Cerrado), Araxá (MG, Cerrado), Tangará da Serra (MT, Cerrado), Cruz Machado (PR, Atlantic Forest), Sertãozinho (SP, Cerrado), São Gotardo (MG, transition between Cerrado and Atlantic Forest), Novo Progresso (PA, Amazon), Ibiá (MG, Cerrado) and Ribeirão Preto (SP, Cerrado). According to our predictions, the risk increases over the years, reaching the maximum risk values in 2015 and 2016, with the maximum values occurring in Brasília, despite it not having the maximum number of cases in the period.

## 4. Discussion

This is the first study predicting hantavirus disease risk and number of cases in humans for the whole of Brazil using landscape, social, climate and biodiversity variables. A key finding of our results is that the amount of forest cover and the rural population at risk positively affect both disease risk and the number of cases. Our results also demonstrated that maize, sugarcane, and temperature can affect hantavirus cardiopulmonary syndrome risk. Our risk map identified that 11% of the country municipalities have a level of risk equal or higher than 5% per year, with 11 municipalities showing a very high risk (>50% risk). High-risk areas were mostly found in Cerrado and Atlantic Forest, followed by Amazon municipalities. This risk increased through time, confirming the emerging characteristic of HCPS in Brazil. The positive influence of both the amount of forest and the number of rural workers seems to explain the high number of cases occurring per year in the central and northern areas of Brazil.

From our hypotheses for explaining disease risk, we found the expected results regarding host diversity, rural workers, sugarcane, and maize amounts. Still, we detected results that differed from expected for temperature, rainfall, forest, pasture and forestry (Figure S1). According to our results, host diversity had a positive effect on the risk of pathogen transmission, but not on the number of human cases.

Disease risk is a complex and local phenomenon that is linked to multiple aspects, including the composition of reservoir host assemblages [54]. In a highly diverse community of rodent species, whose distributions overlap with a great number of other rodent species, a high viral diversity is expected [55]. HCPS in Brazil is caused by ARQV, JUQV, CASV, LANV, RIOMV and ANJV [21]. Different genotypes and strains, such as JUQV, ARQV and CASV belong to ANDV clade, while LANV, ANJV and RIOMV belong to LANV clade [21]. However, despite the diversity, the “viral load” in the communities may be very low, if there is “dilution” through high viral host species’ contacts limiting viral transmission among heterogeneously susceptible host species, lowering overall transmission risk to humans. There is some evidence that mammalian biodiversity is the best predictor of zoonotic disease diversity at a large scale, but not of specific diseases [25], and here we show this effect must be further investigated, because we found a positive effect of host diversity on HCPS risk. We hypothesize that this happened because a high diversity of hosts is related to a greater diversity of viruses circulating in the same region and, despite the low viral load—if there is a certain threshold rodent population abundance in order to maintain the virus in the environment—disease risk could be increased. However, if more than one main rodent species can effectively transmit viral lineages, the mechanism for the dilution effect (heterogeneous host susceptibility) will be less strong, and local susceptible host abundance will be less affected by species diversity, limiting the impact of species diversity on disease risk. Further, hantavirus particles may remain infectious for weeks, depending on temperature, humidity, and association with protective proteins [23], thus limiting the impact of increasing host diversity on intra-species transmission.

Our results show that the effect size of the population at risk is the highest among the risk factors for disease risk. This finding corroborates the occupational nature of hantavirus disease in Brazil and that rural activities define the risk, as has been reported for other regions [21]. The positive effect of rural worker population sizes on disease risk was expected and found for the Atlantic Forest biome [10], but not previously for Cerrado areas [10,56]. Notwithstanding overall rural worker populations decline through time, the disease risk still increased and was positively influenced by the rural worker populations in municipalities. This fact highlights the need for better prevention measures in situ.

In a multicriteria analysis using expert knowledge, it was pointed out that areas such as Mato Grosso state (MT) had increased disease vulnerability, and the risk might be increasing in areas of previous disease “absence”, such as the northern region of Brazil [6]. Indeed, we verified that the overall risk increased with time (Figure S5a) although we notice a trend towards a reduction in the overall number of cases over time. This indicates a geographical expansion of the disease, which is increasingly detected in more municipalities.

The positive effect of forest amount on disease risk was not expected, but should be further investigated, since when we look at the entire country we have different host species with different habits and habitat requirements [19,20]. It is notable how the disease reports increased in northern areas of Brazil recently, in areas that have high native forest cover. In this area, the effect of forest amount on hantavirus disease risk was positive and consistent. Those areas with increased risk might be where there is contact between forested areas and rural areas where both rodents and workers transit through.

We also found a positive effect of sugarcane expansion on disease risk, but not for the total number of cases. Sugarcane expanded a lot from 2000–2010 (Figure S3), but so did machinery technology and the cessation of pre-harvest fires in some locations [57]. Moreover, worker conditions are expected to vary across different states, and the positive effect of sugarcane and host richness seem to be concentrated in Atlantic Forest and Cerrado areas in southeastern Brazil (Figure S8), while other conditions impose more influence in the total number of disease cases in the rest of the country, such as the amount of forest, rainfall and rural population numbers. Another hypothesis is that there is a time lag between the effects of sugarcane expansion and the influence of land use alteration on the number of cases, meaning there will be a detectable problem in the future if prevention measures are not applied. The potential effect of pasture on hantavirus disease risk through the availability of grasses, such as brachiaria, for rodent hosts that can locally benefit from that may be present, but it was not detected in our study. Confounding associations, such as the presence or intensity of land use for cattle production, could limit the local suitability for hosts along with our ability to make inferences regarding risk from pasture areas. More local analyses may find disease risk is increased in these areas.

With respect to the long-term data, we had intrinsic limitations to model risk due to the rarity of notified cases in the first years of the time series (1993–1999). Moreover, we had the limitation of linearity among sparse data. It is important to investigate the possible non-linearity of effects, since we have many areas composed almost entirely by rural work forces in the Northeast of Brazil that have never had hantavirus disease cases notified and clearly are not suitable for the known hosts of hantavirus (Figure 3). Therefore, the large effect of the rural population at risk must be carefully interpreted, bearing in mind that there are localities in Brazil that lack the hosts, and thus might make the probability of the disease non-realistic.

Regarding spatial clustering of high-risk areas, we highlight that we found spatial random effects higher than 8%. Those areas are municipalities where attention has been given to hantavirus disease surveillance. Detecting these effects becomes even more important to support government strategies that focus on the control of the disease in Brazil and to elucidate if current government action measures are being effective to prevent the hantavirus expansion. Despite the increase in disease risk, it seems that the trend in outbreaks (i.e., case counts) decreased through time, which could be a consequence of specific applications of site monitoring or improvement of working conditions and guidance on safety at work (personal protective equipment, mechanization or improvement in working protocols). We did not include those variables in our models, but there are government efforts to prevent hantavirus disease in Brazil, such as the “Manual of surveillance, prevention and control of hantavirus cardiopulmonary disease” published in 2014 [12] and local monitoring from federal and state government Institutes, such as FIOCRUZ and Adolfo Lutz.

In conclusion, we showed that host diversity, social vulnerability, climate, and land use change influence hantavirus disease risk in Brazil. The expansion of sugarcane and maize plantations lead to an increase in HCPS disease risk, with additive positive effects from social vulnerability (despite an evident rural exodus trend) and native forest amount in a municipality. Confirmed cases are useful to understand the disease transmission risk of this relatively rare disease because case occurrences are provided by Brazilian Ministry of Health in unified databases of communicable diseases. Despite compulsory but suboptimal notification and diagnostic difficulties for HCPS, these datasets are the most reliable data available. Modeling approaches that maximize the predictive efficiency while reducing the computational time or field sampling effort are desired to predict emerging diseases. We recommend that HCPS mitigation and surveillance strategies need to be applied to prevent future outbreaks in the following highest-risk municipalities and their surroundings: Brasília, Campo Novo dos Parecis, Patrocínio, Araxá, Tangará da Serra, Cruz Machado, Sertãozinho, São Gotardo, Novo Progresso, Ibiá and Ribeirão Preto.

## Supplementary Materials

The following are available online at https://www.mdpi.com/1999-4915/11/11/1008/s1, GIF S1: Spatiotemporal dynamics of hantavirus cardiopulmonary syndrome risk in Brazilian municipalities. Figure S1: Coefficient plot based on our working hypothesis for hantavirus disease risk in Brazil. Negative coefficients represent a decrease in risk, positive values represent an increase in risk. Expected effect sizes were inferred from previous findings [6,10,11,58]. Figure S2: Pair plot and correlation between predictors used to model hantavirus disease risk in Brazil. Values correspond to Pearson´s correlation coefficients. Figure S3: Fixed covariate values averaged over municipalities. We used the covariates in the modeling procedure of hantavirus disease numbers and the probability of cases in Brazil from 2000–2016. Host richness was considered constant along time. Figure S4: Hantavirus disease distribution in Brazil. Current expected values for the probability of hantavirus disease in humans, predicted by a spatiotemporal model containing forest, climate, and population at risk. Top five municipalities in terms of risk per year are highlighted. See the risk map with the uncertainty layer in Figure 3. Figure S5: (a) Time trend for a binomial model estimating the probability of hantavirus disease cases in humans as a function of biodiversity, climate, social vulnerability and landscape change between 2000 to 2014 in Brazil. (b) Predictions for 2015 and 2016 using new data for covariates and NA data for response variable. Figure S6: (a) Time trend for a zero truncated Poisson model estimating the number of cases in humans as a function of biodiversity, climate, social vulnerability and landscape change between 2000 to 2014 in Brazil. (b) Predictions for 2015 and 2016 using new data for covariates and NA data for response variable. Figure S7: Municipalities showing positive effects of the spatial random field on hantavirus disease risk. The municipalities in labels are the ones with top five higher spatial random effect values. Figure S8: The number of species of hosts and the sugarcane amount in the municipality. The bubble size is proportional to the average predicted HCPS risk. Points are colored according to biome. Table S1: Data sources used in the spatiotemporal modelling procedure. Table S2: Spatial auto-correlation tests for the distribution of hantavirus disease cases in humans in Brazil. Using 5% as alpha error level, we observe some clustering in 2006 and 2013. sd = standard deviation. In 1994 and 1997 no cases were notified. Table S3: Rodent host selected predictors in models based on climate. Factorial analysis showing which factors contribute more for the variation in multivariate axes over the extent. This analysis helps select the most meaningful variables for explaining the environmental gradient that possibly correlates with species distribution. Factorial analysis uses correlation among input variables to sort related variables into “Factors”. From this analysis you can pick the ones that contribute more for each factor as a suitable variable describing the climatic patterns from all variables that are available to use. Selected variables in boldface. Table S4: Performance of suitable niche models for explaining host occurrence in Brazil. After model selection (TSS > 0.5) we used expert opinion to validate the 10-percentile threshold for observed presence data to infer presence. Table S5: Fixed covariate averages for 5570 municipalities of Brazil from 2000–2016. Table S6: Model estimates from the selected predictors of hantavirus disease in humans in Brazil. Those values are related to Figure 5 in the main text. Table S7: Average hantavirus disease risk in municipalities of Brazil with risk equal to or greater than 5%, in decreasing order, considering a Bernoulli model for the period from 2000–2014 for all 5570 municipalities of Brazil. Municipality contains the Municipality code followed by municipality name without special characters. Risk was rounded to 3 decimal places.
