# Supplementary material for: Spatiotemporal Dynamics of Hantavirus Cardiopulmonary Syndrome Transmission Risk in Brazil
Source: Viruses. 2019 Oct 31;11(11):1008. doi: 10.3390/v11111008 (PMC6893581; doi:10.3390/v11111008)
Supplement: Supplementary file 1 [file viruses-11-01008-s001.zip › viruses-631319-for conversion final/for conversion-supplementary.pdf]

Supplementary Materials

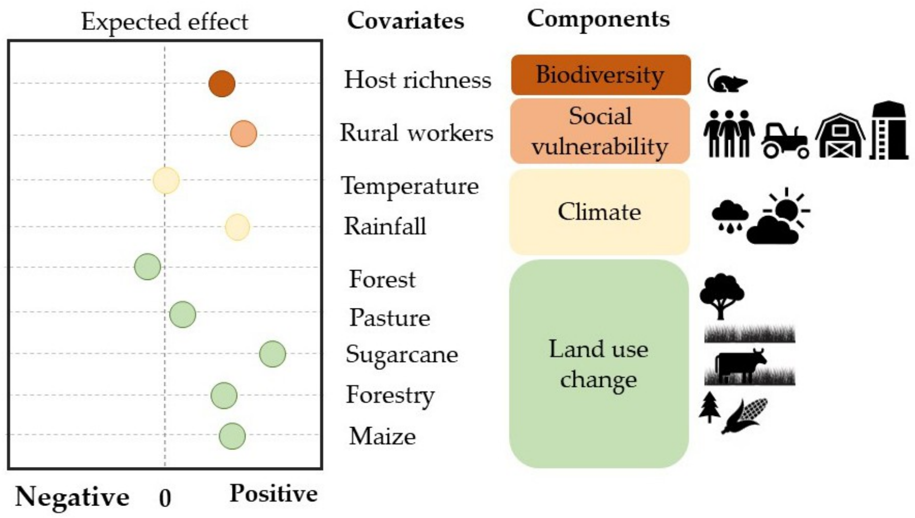

Figure S1. Coefficient plot based on our working hypothesis for hantavirus disease risk in Brazil. Negative coefficients represent a decrease in risk, positive values represent an increase in risk. Expected effect sizes were inferred from previous findings [6,10,11,24].

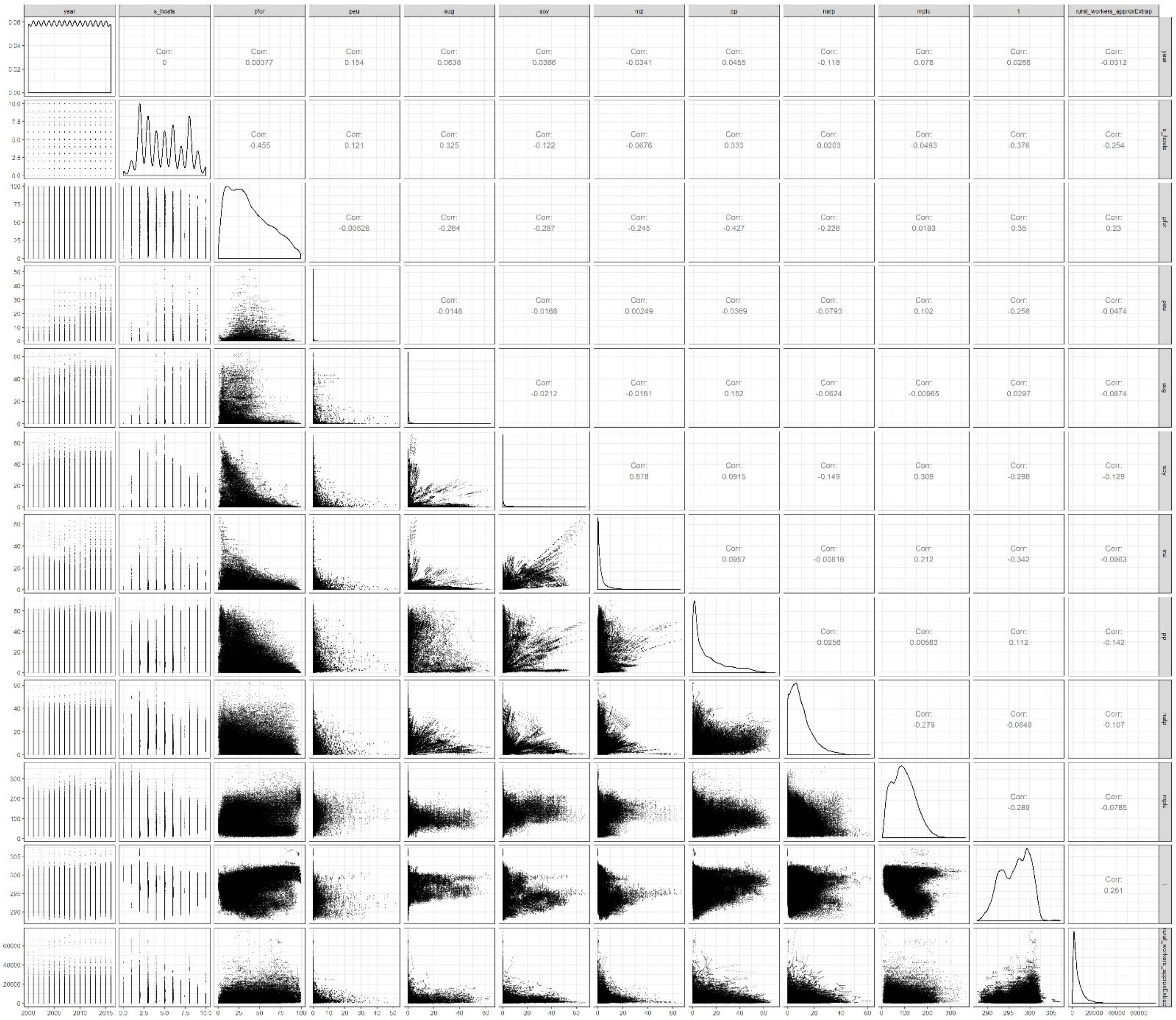

Figure S2. Pair plots and correlation between predictors used to model hantavirus disease risk in Brazil. Values correspond to Pearson´s correlation coefficients.

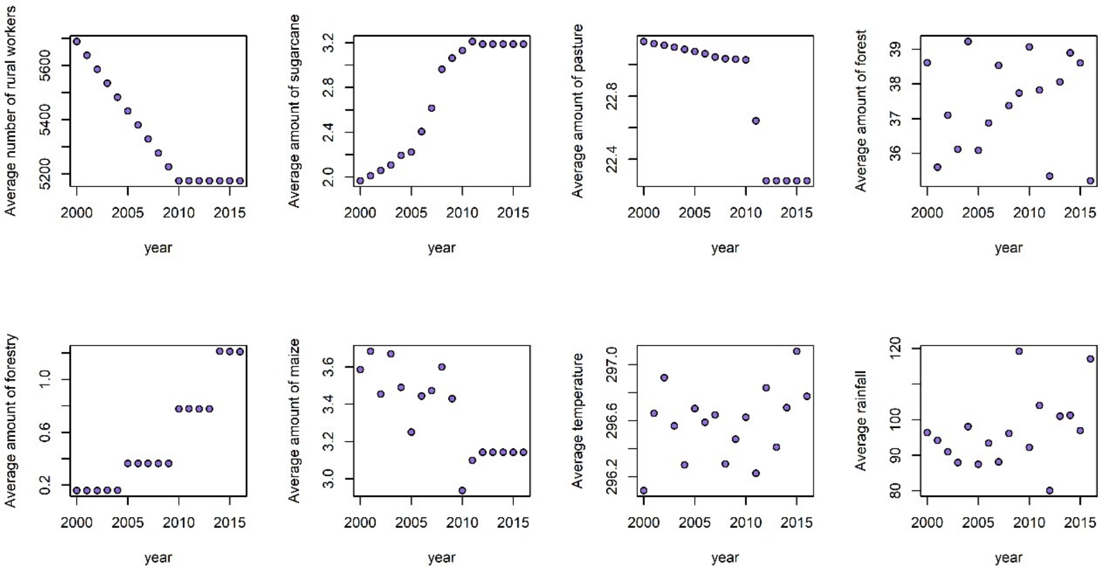

Figure S3. Fixed covariate values averaged over municipalities. We used the covariates in the modeling procedure of hantavirus disease numbers and the probability of cases in Brazil from 2000-2016. Host richness was considered constant through time.

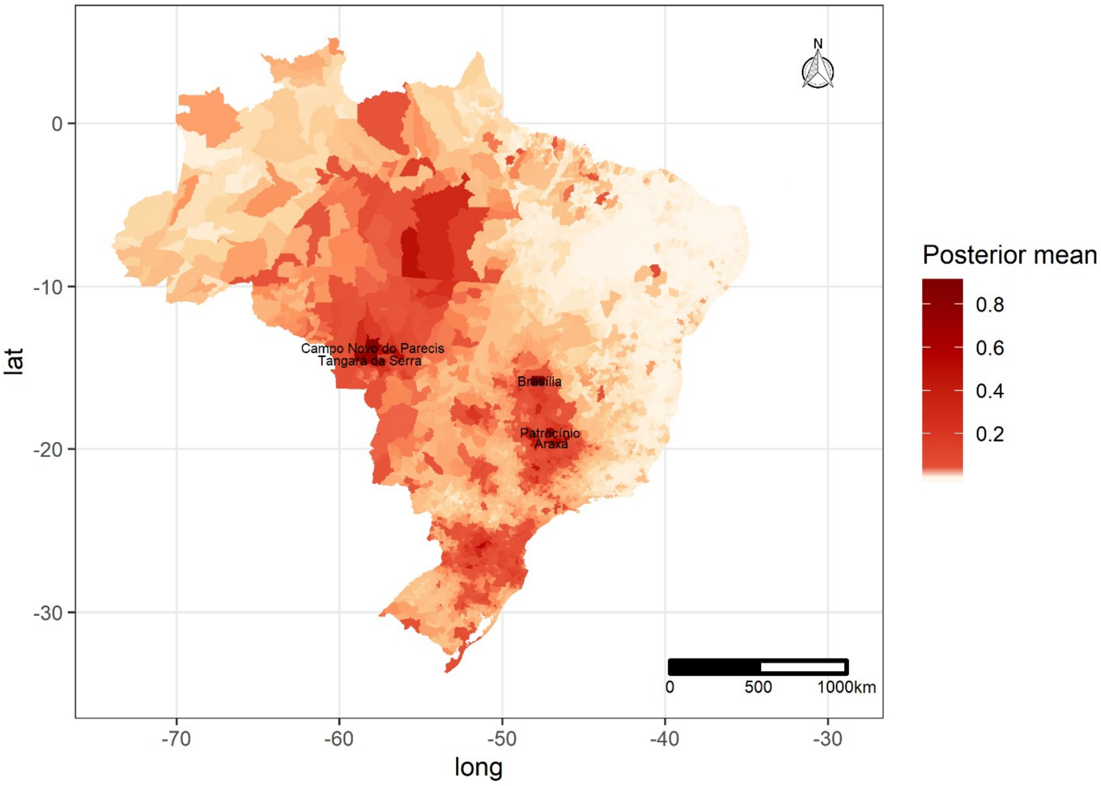

Figure S4. Hantavirus disease distribution in Brazil. Current expected values for the probability of hantavirus disease in humans, predicted by a spatio-temporal model containing forest, climate, and population at risk. Top five municipalities in terms of risk per year are highlighted. See the risk map with the uncertainty layer in Figure 3.

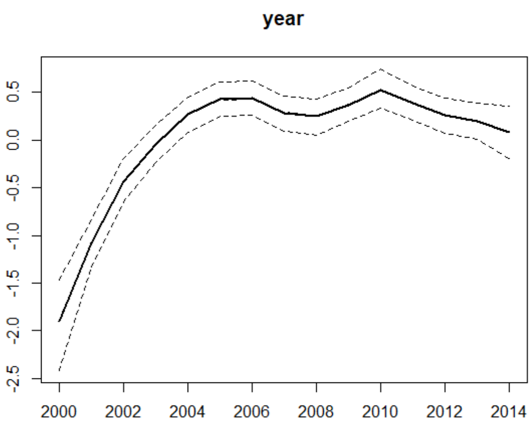

(a)

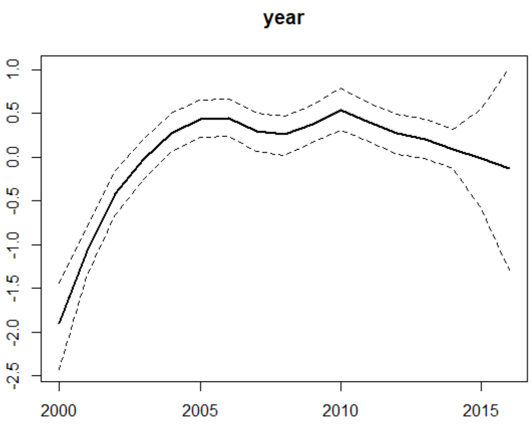

(b)

Figure S5. (a) Time trend for a binomial model estimating the probability of hantavirus disease cases in humans as a function of biodiversity, climate, social vulnerability and landscape change between 2000 to 2014 in Brazil. The y axis represents random effect values and the predictor is each year**.** (b) Predictions for 2015 and 2016 using new data for covariates and NA data for response variable. Dashed lines correspond to 95% credible intervals or the posterior mean.

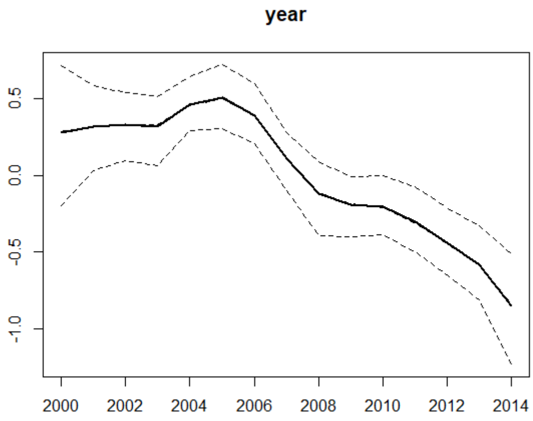

(a)

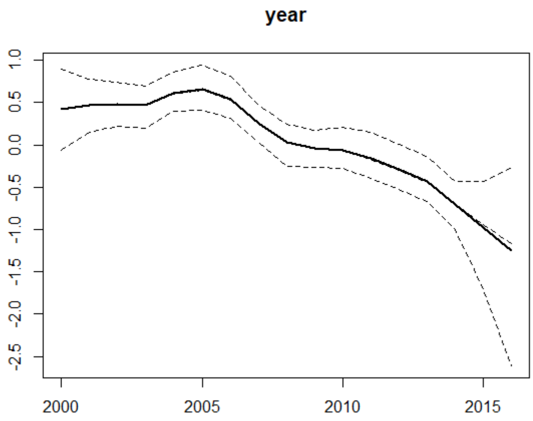

(b)

Figure S6. (a) Time trend for a zero truncated Poisson model estimating the number of cases in humans as a function of biodiversity, climate, social vulnerability and landscape change between 2000 to 2014 in Brazil. The y axis represents random effect values and the predictor is each year**.** (b) Predictions for 2015 and 2016 using new data for covariates and NA data for response variable. Dashed lines correspond to 95% credible intervals or the posterior mean.

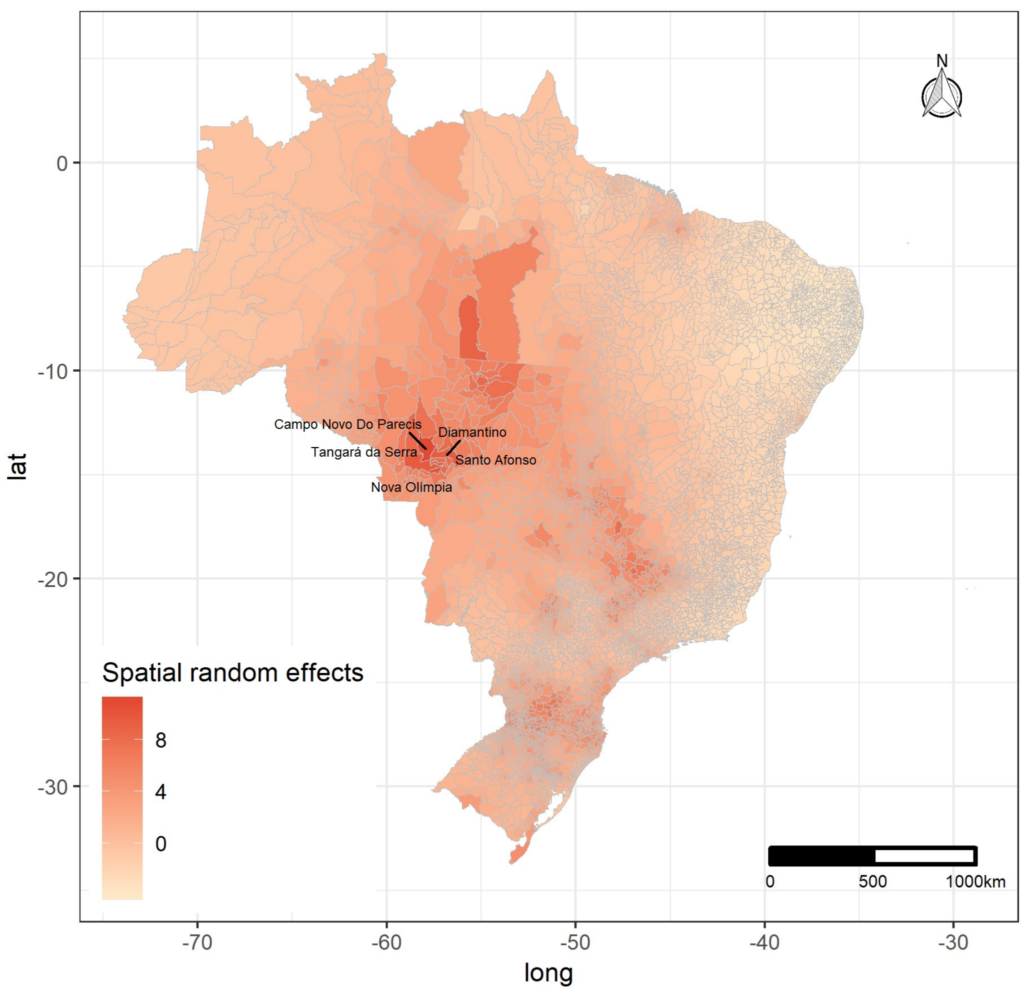

Figure S7. Municipalities showing positive effects of the spatial random field on hantavirus disease risk. The municipalities in labels are the ones with top five higher spatial random effect values.

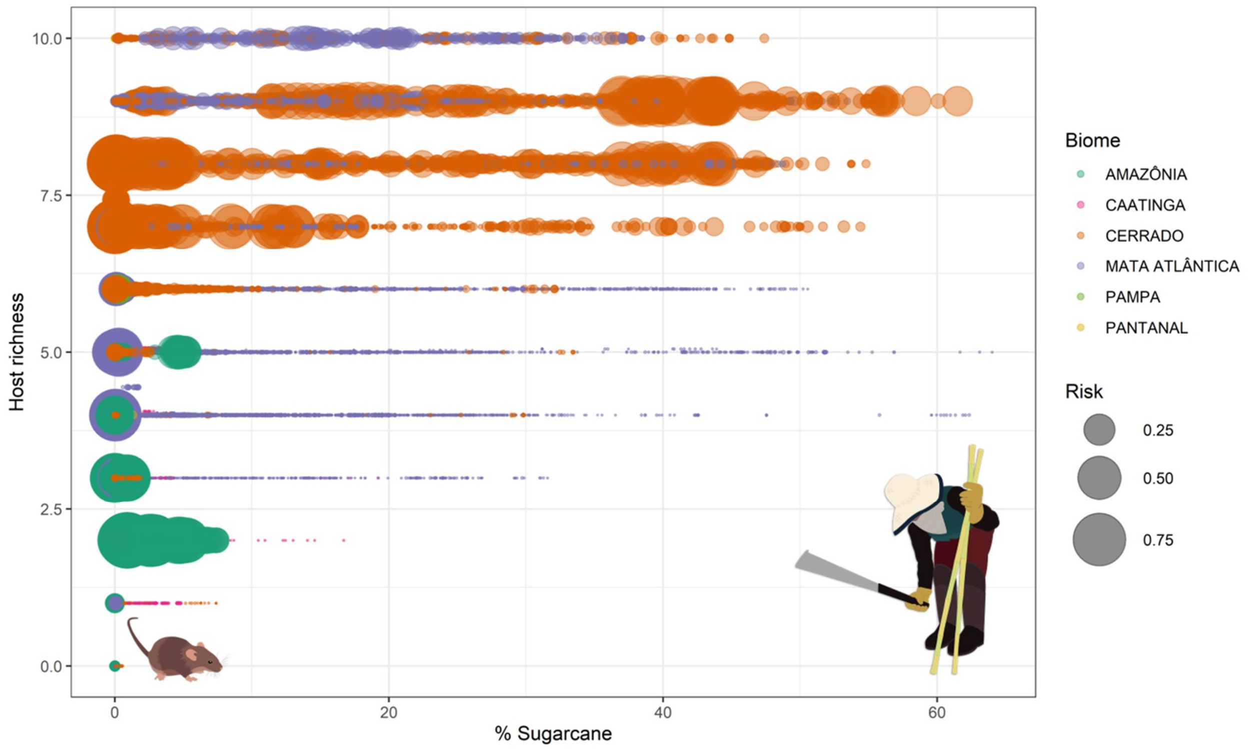

Figure S8. The number of species of hosts and the sugarcane amount in the municipality. The bubble size is proportional to the average predicted HCPS risk. Points are colored according to biome.

Table S1. Data sources used in the spatial-temporal modelling procedure.

Variable	Time span	Spatial resolution in decimal degrees (Metric)/ Cartographic scale (Metric)	Source	Source URL
Surface Temperature	2000-2016	0.5 (50 km)	NOAA NCEP	iridl.ldeo.columbia.edu/SOURCES/.NOAA/, http://iridl.ldeo.columbia.edu/SOURCES/.NOAA/.NCEP/.CPC/.GHCN_CAMS/.gridded/.deg0p5/.temp/
Rainfall	2000-2016	0.05 (5 km)	CHIRPS	http://chg.geog.ucsb.edu/data/chirps/, https://pubs.usgs.gov/ds/832/
Agricultural land use	2000-2014	0.01 (1 km)	Dias et al 2016 GCB	biosfera.dea.ufv.br/pt-BR/banco/uso-do-solo-agricola-no-brasil-1940-2012---dias-et-al-2016
Native habitat pasture	2000-2014	0.01 (1 km)	Dias et al 2016 GCB	biosfera.dea.ufv.br/pt-BR/banco/uso-do-solo-agricola-no-brasil-1940-2012---dias-et-al-2016
Native habitat forests and forestry	2000-2016	0.0003 (30 m)	Mapbiomas2	http://mapbiomas.org/pages/database/mapbiomas_collection
Political divisions	2015	1:500000 (2.5 km)	IBGE	ftp://geoftp.ibge.gov.br/organizacao_do_territorio/malhas_territoriais/malhas_municipais/
Hantavirus cases in humans	1993-2017	1:500000 (5 km)	M Health	datasus.gov.br
Census	1991, 2000, 2006, 2010	1:500000 (5 km)	IBGE	https://downloads.ibge.gov.br/downloads_estatisticas.htm, ATLAS ONU, ftp://ftp.ibge.gov.br/Estimativas_de_Populacao/, ftp://ftp.ibge.gov.br/Censos/
Biome limits	2004	1:250000 (2.5 km)	MMA	http://mapas.mma.gov.br/i3geo/datadownload.htm, https://drive.google.com/file/d/0Byp5eRWoQ-Pka0hITTVDVWVfSFU/view?ts=5984ccbf

Table S2. Spatial auto-correlation tests for the distribution of hantavirus disease cases in humans in Brazil. Using 5% as alpha error level, we observe some clustering in 2006 and 2013. sd= standard deviation. In 1994 and 1997 no cases were notified.

Year	Observed	Expected	sd	P value
1993	-0.0001	-0.0002	0.0001	0.4953
1995	-0.0002	-0.0002	0.0001	0.4571
1996	-0.0003	-0.0002	0.0046	0.9864
1998	0.0008	-0.0002	0.0051	0.8494
1999	-0.0008	-0.0002	0.005	0.8988
2000	0.001	-0.0002	0.0046	0.806
2001	0.0041	-0.0002	0.0053	0.4121
2002	0.0034	-0.0002	0.0053	0.4963
2003	0.0035	-0.0002	0.0055	0.5015
2004	0.0018	-0.0002	0.0042	0.6334
2005	0.0028	-0.0002	0.0052	0.5711
2006	0.0119	-0.0002	0.0052	0.0207
2007	0.0006	-0.0002	0.0054	0.8872
2008	0.0052	-0.0002	0.0049	0.2795
2009	0.0022	-0.0002	0.0052	0.6422
2010	0.0024	-0.0002	0.0047	0.5884
2011	0.0052	-0.0002	0.0054	0.3223
2012	0.0073	-0.0002	0.0055	0.1729
2013	0.0109	-0.0002	0.0052	0.0332
2014	0.0044	-0.0002	0.0055	0.4026
2015	0.0032	-0.0002	0.0054	0.5332
2016	0.0019	-0.0002	0.0051	0.6759

Table S3. Rodent host selected predictors in models based on climate. Factorial analysis showing which factors contribute more for the variation in multivariate axes over the extent. This analysis helps select the most meaningful variables for explaining the environmental gradient that possibly correlates with species distribution. Factorial analysis uses correlation among input variables to sort related variables into "Factors". From this analysis you can pick the ones that contribute more for each factor as a suitable variable describing the climatic patterns from all variables that are available to use. Selected variables in boldface.

Bioclimatic variable	MR1	MR2	MR3	MR6	MR4	MR5
BIO1 = Annual Mean Temperature	0.94	-0.06	0.23	-0.15	0.18	-0.06
**BIO2 = Mean Diurnal Range (Mean of monthly (max temp - min temp))**	-0.13	-0.40	-0.05	**0.89**	0.00	0.13
**BIO3 = Isothermality (BIO2/BIO7) (* 100)**	0.56	0.14	0.30	-0.26	**0.66**	-0.04
BIO4 = Temperature Seasonality (standard deviation *100)	-0.64	0.12	-0.43	0.16	-0.51	0.07
BIO5 = Max Temperature of Warmest Month	0.88	-0.17	0.17	0.32	-0.12	-0.20
BIO6 = Min Temperature of Coldest Month	0.79	0.12	0.24	-0.44	0.28	-0.17
BIO7 = Temperature Annual Range (BIO5-BIO6)	-0.42	-0.25	-0.19	0.74	-0.42	0.09
BIO8 = Mean Temperature of Wettest Quarter	0.87	-0.13	0.13	-0.11	0.11	0.15
BIO9 = Mean Temperature of Driest Quarter	0.83	0.03	0.28	-0.21	0.23	-0.26
**BIO10 = Mean Temperature of Warmest Quarter**	**0.97**	0.01	0.11	-0.15	-0.06	-0.12
BIO11 = Mean Temperature of Coldest Quarter	0.88	-0.07	0.31	-0.16	0.30	-0.10
BIO12 = Annual Precipitation	0.22	0.55	0.77	-0.07	0.09	0.14
BIO13 = Precipitation of Wettest Month	0.29	0.04	0.93	-0.07	0.11	0.02
BIO14 = Precipitation of Driest Month	-0.06	0.95	0.11	-0.17	0.10	0.09
BIO15 = Precipitation Seasonality (Coefficient of Variation)	0.19	-0.87	-0.01	0.13	0.15	-0.11
BIO16 = Precipitation of Wettest Quarter	0.30	0.07	0.94	-0.05	0.10	0.05
**BIO17 = Precipitation of Driest Quarter**	-0.06	**0.97**	0.13	-0.17	0.09	0.10
**BIO18 = Precipitation of Warmest Quarter**	-0.26	0.36	0.15	0.19	-0.04	**0.79**
BIO19 = Precipitation of Coldest Quarter	0.22	0.48	0.45	-0.37	0.12	-0.27

Table S4. Performance of suitable niche models for explaining host occurrence in Brazil. After model selection (TSS>0.5) we used expert opinion to validate the 10-percentile threshold for observed presence data to infer host presence.

Species	Mean AUC	Mean TSS	SD AUC	SD TSS	Hantavirus genotypes
Akodon cursor	0.82	0.62	0.11	0.18	JUQV JUQV like
Akodon montensis	0.92	0.79	0.07	0.14	ARQV JABV
Calomys tener	0.88	0.77	0.08	0.14	ARQV
Holochilus sciureus	0.55	0.34	0.21	0.26	RIOMM
Necromys lasiurus	0.7	0.41	0.07	0.11	ARQV
Oligoryzomys eliurus	0.55	0.35	0.23	0.27	CASV
Oligoryzomys fornesi	0.82	0.64	0.13	0.24	ANJV
Oligoryzomys microtis	0.52	0.32	0.26	0.29	RIOMV
Oligoryzomys nigripes	0.86	0.67	0.07	0.12	JUQV, ARQV
Oxymycterus dasytrichus	0.85	0.7	0.14	0.23	JUQV

Table S5. Fixed covariate averages for 5570 municipalities of Brazil from 2000-2016.

Year	2000	2001	2002	2003	2004	2005	2006	2007	2008	2009	2010	2011	2012	2013	2014	2015	2016
**Rural workers**	5688.8	5637.44	5586.09	5534.73	5483.38	5432.02	5380.67	5329.31	5277.96	5226.61	5175.25	5175.25	5175.25	5175.25	5175.25	5175.25	5175.25
**min**	62	62	61	61	61	61	61	61	60	60	60	60	60	60	60	60	60
**max**	66055	65730	65405	65080	64755	64430	65928	68128	70327	72526	74725	74725	74725	74725	74725	74725	74725
**Sugarcane (%)**	1.97	2.01	2.06	2.11	2.19	2.22	2.41	2.61	2.96	3.06	3.13	3.21	3.19	3.19	3.19	3.19	3.19
**min**	0	0	0	0	0	0	0	0	0	0	0	0	0	0	0	0	0
**max**	64	63.09	61.66	60.25	60.48	60.69	60.96	61.32	61.88	62.05	62.34	56.18	56.1	56.1	56.1	56.1	56.1
**Pasture (%)**	23.15	23.13	23.12	23.11	23.1	23.08	23.07	23.05	23.04	23.03	23.03	22.64	22.26	22.26	22.26	22.26	22.26
**min**	0	0	0	0	0	0	0	0	0	0	0	0	0	0	0	0	0
**max**	86.9	85.76	86.54	87.7	88.86	90.03	91.33	92.5	93.69	95.94	101	91.91	83.93	83.93	83.93	83.93	83.93
**Forest (%)**	38.61	35.6	37.1	36.12	39.23	36.09	36.87	38.54	37.38	37.74	39.07	37.83	35.34	38.06	38.9	38.61	35.21
**min**	0	0	0	0	0	0	0	0	0	0	0	0	0	0	0	0	0
**max**	99.55	99.31	99.32	99.41	99.41	99.56	99.56	99.54	99.55	99.2	99.16	99.53	99.53	99.48	99.36	99.47	99.01
**Forestry (%)**	0.16	0.16	0.16	0.16	0.16	0.36	0.36	0.36	0.36	0.36	0.78	0.78	0.78	0.78	1.21	1.21	1.21
**min**	0	0	0	0	0	0	0	0	0	0	0	0	0	0	0	0	0
**max**	23.17	23.17	23.17	23.18	23.18	35.38	35.37	35.36	35.4	35.38	45.29	45.34	45.26	45.18	51.79	51.78	51.75
**Maize**	3.59	3.69	3.45	3.67	3.49	3.25	3.44	3.47	3.6	3.43	2.94	3.1	3.14	3.14	3.14	3.14	3.14
**min**	0	0	0	0	0	0	0	0	0	0	0	0	0	0	0	0	0
**max**	55.66	57.83	56.7	61.71	60.76	47.44	53.23	58.26	62.86	62.12	54.89	62.87	65.96	65.96	65.96	65.96	65.96
**Temperature (10*°C)**	296.1	296.65	296.91	296.56	296.28	296.69	296.59	296.64	296.29	296.47	296.62	296.22	296.84	296.41	296.69	297.1	296.77
**min**	287.68	288.78	288.65	288.18	287.77	288.41	288.39	288.17	287.8	288.04	288.03	287.79	288.76	287.7	288.82	288.81	287.87
**max**	306.5	306.89	306.89	306.8	306.82	306.82	306.41	306.63	306.27	307.11	306.36	306.38	306.61	306.69	305.98	306.35	306.44
**Rainfall (mm/month)**	96.37	94.14	90.97	87.93	98.01	87.44	93.43	88.15	96.11	119.23	92.19	103.95	80.08	100.98	101.21	96.9	117.05
**min**	12	8	10	11	10	8	6	8	8	15	10	9	4	9	8	7	8
**max**	296	267	326	265	266	280	332	288	314	330	324	283	286	302	304	285	364

Table S6. Model estimates from the selected predictors of hantavirus disease in humans in Brazil. Those values are related to Figure 5 in the main text. PA = presence/absence of cases, Cases = number of cases.

Response variable	Component	Coefficient	Model	Mean	95% Credible interval
	-	Intercept	Binomial with logit link	-8.347	-8.875 to -7.893
PA	Biodiversity	Host richness	Binomial with logit link	0.797	0.413 to 1.185
PA	Social	Population at risk	Binomial with logit link	0.777	0.668 to 0.886
PA	Climate	Rainfall	Binomial with logit link	0.071	-0.075 to 0.216
PA	Climate	Temperature	Binomial with logit link	-0.528	-0.968 to -0.088
PA	Land use change	Forest	Binomial with logit link	0.29	0.064 to 0.514
PA	Land use change	Pasture	Binomial with logit link	-0.08	-0.31 to 0.147
PA	Land use change	Sugarcane	Binomial with logit link	0.182	0.002 to 0.361
PA	Land use change	Forestry	Binomial with logit link	-0.082	-0.168 to -0.0006
PA	Land use change	Maize	Binomial with logit link	0.159	0.002 to 0.310
Cases	-	Intercept	Zero-Truncated poisson with log link	-0.988	-1.726 to -0.304
Cases	Biodiversity	Host richness	Zero-Truncated poisson with log link	0.005	-0.410 to 0.426
Cases	Social	Population at risk	Zero-Truncated poisson with log link	0.366	0.219 to 0.513
Cases	Climate	Rainfall	Zero-Truncated poisson with log link	0.111	-0.007 to 0.229
Cases	Climate	Temperature	Zero-Truncated poisson with log link	-0.208	-0.716 to 0.299
Cases	Land use change	Forest	Zero-Truncated poisson with log link	0.262	0.023 to 0.502
Cases	Land use change	Pasture	Zero-Truncated poisson with log link	0.036	-0.249 to 0.320
Cases	Land use change	Sugarcane	Zero-Truncated poisson with log link	0.102	-0.279 to 0.474
Cases	Land use change	Forestry	Zero-Truncated poisson with log link	0.04	-0.111 to 0.181
Cases	Land use change	Maize	Zero-Truncated poisson with log link	0.015	-0.19 to 0.217

Table S7. Average hantavirus disease risk in municipalities of Brazil with risk equal to or greater than 5%, in decreasing order, considering a Bernoulli model for the period from 2000-2014 for all 5570 municipalities of Brazil. Municipality contains the Municipality code followed by municipality name without special characters. Risk was rounded to 3 decimal places. Data for all municipalities per year can be downloaded at https://github.com/renatamuy/Muylaert_et_al_2019

Municipality	Risk
530010 Brasilia	0.771
510263 Campo Novo do Parecis	0.749
314810 Patrocinio	0.727
310400 Araxa	0.671
510795 Tangara da Serra	0.643
410680 Cruz Machado	0.616
355170 Sertaozinho	0.602
316210 Sao Gotardo	0.579
150503 Novo Progresso	0.555
312950 Ibia	0.521
354340 Ribeirao Preto	0.506
420430 Concordia	0.493
510623 Nova Olimpia	0.481
510350 Diamantino	0.479
317020 Uberlandia	0.441
317010 Uberaba	0.439
412200 Rio Azul	0.439
354020 Pontal	0.436
410850 General Carneiro	0.426
410290 Bituruna	0.381
316510 Sao Tomas de Aquino	0.351
520480 Campo Alegre de Goias	0.333
150060 Altamira	0.330
314980 Perdizes	0.324
411390 Mallet	0.312
420770 Ipumirim	0.311
412820 Uniao da Vitoria	0.309
510642 Peixoto de Azevedo	0.305
411020 Inacio Martins	0.304
420160 Arroio Trinta	0.293
354890 Sao Carlos	0.293
520110 Anapolis	0.289
510805 Terra Nova do Norte	0.287
420240 Blumenau	0.285
315550 Rio Paranaiba	0.276
421930 Videira	0.265
311150 Campos Altos	0.255
411070 Irati	0.254
510726 Santo Afonso	0.253
411440 Mangueirinha	0.251
315050 Pimenta	0.250
510787 Sapezal	0.233
510170 Barra do Bugres	0.232
411930 Pinhao	0.229
420900 Joacaba	0.229
420070 Alfredo Wagner	0.227
410940 Guarapuava	0.220
350320 Araraquara	0.218
412030 Porto Vitoria	0.213

522185 Valparaiso de Goias	0.212
314800 Patos de Minas	0.205
351930 Ibate	0.203
420985 Lindoia do Sul	0.203
510558 Marcelandia	0.201
510730 Sao Jose do Rio Claro	0.199
352370 Itirapua	0.199
411760 Palmas	0.197
410645 Coronel Domingos Soares	0.196
421590 Sao Bonifacio	0.195
316810 Tapira	0.193
521190 Jatai	0.193
420850 Ituporanga	0.193
421260 Peritiba	0.191
420127 Arabuta	0.190
150680 Santarem	0.189
350560 Barrinha	0.189
420300 Cacador	0.188
421003 Luzerna	0.188
420980 Leoberto Leal	0.179
421780 Taio	0.178
316220 Sao Joao Batista do Glaria	0.177
314790 Passos	0.174
420860 Jabora	0.170
421935 Vitor Meireles	0.169
353430 Orlandia	0.168
510190 Brasnorte	0.168
410400 Campina Grande do Sul	0.168
412060 Prudentopolis	0.167
311510 Cassia	0.167
421750 Seara	0.165
412560 Sao Mateus do Sul	0.163
510320 Colider	0.162
520620 Cristalina	0.162
421875 Tunapolis	0.159
431680 Santa Cruz do Sul	0.159
421450 Rio do Campo	0.158
421570 Santo Amaro da Imperatriz	0.158
353060 Mogi das Cruzes	0.158
521250 Luziania	0.157
355030 Sao Paulo	0.156
351090 Cassia dos Coqueiros	0.154
420765 Ipora do Oeste	0.152
421360 Porto Uniao	0.152
351460 Dumont	0.150
421370 Pouso Redondo	0.149
420680 Ibicare	0.145
420810 Itaiopolis	0.145
421670 Sao Jose do Cedro	0.145
420757 Iomere	0.142
521975 Santo Antonio do Descoberto	0.141
316680 Serra do Salitre	0.141
421920 Vidal Ramos	0.140
510340 Cuiaba	0.139
510600 Nortelandia	0.136
510560 Matupa	0.135

420090 Angelina	0.134
510345 Denise	0.133
316470 Sao Sebastiao do Paraiso	0.133
421470 Rio dos Cedros	0.130
313750 Lagoa Formosa	0.130
421020 Major Gercino	0.128
420670 Herval d'Oeste	0.127
430510 Caxias do Sul	0.126
421220 Papanduva	0.126
315150 Piumhi	0.125
315770 Santa Juliana	0.125
312890 Guimarania	0.125
421430 Rancho Queimado	0.125
420010 Abelardo Luz	0.124
351310 Cravinhos	0.124
520549 Cidade Ocidental	0.124
421960 Xavantina	0.122
420420 Chapeco	0.122
315690 Sacramento	0.119
421500 Rio Negrinho	0.119
510626 Novo Mundo	0.119
310740 Bom Despacho	0.119
421340 Ponte Serrada	0.117
510619 Nova Santa Helena	0.116
420190 Aurora	0.115
510885 Nova Marilandia	0.115
310070 Agua Comprida	0.115
510410 Guaranta do Norte	0.114
520840 Goianapolis	0.114
311930 Coromandel	0.111
313160 Irai de Minas	0.111
350590 Batatais	0.111
314650 Pains	0.109
353010 Mirandopolis	0.109
421085 Mirim Doce	0.108
520870 Goiania	0.107
421567 Santa Terezinha	0.107
420260 Bom Retiro	0.105
354760 Santa Rosa de Viterbo	0.104
351860 Guariba	0.104
421410 Presidente Nereu	0.103
431238 Monte Belo do Sul	0.103
420640 Guaraciaba	0.102
420775 Iraceminha	0.102
314320 Monte Santo de Minas	0.102
520510 Catalao	0.101
430440 Canela	0.101
420780 Irani	0.100
310510 Bambui	0.100
150730 Sao Felix do Xingu	0.100
421480 Rio do Sul	0.099
421420 Quilombo	0.099
412080 Quatro Barras	0.097
314700 Paracatu	0.097
420740 Imbuia	0.097
421540 Salto Veloso	0.097

421390 Presidente Castello Branco	0.096
421530 Salete	0.095
510130 Arenapolis	0.095
310420 Arcos	0.093
421850 Treze Tilias	0.093
315300 Pratinha	0.091
412175 Reserva do Iguacu	0.091
412150 Reboucas	0.091
420380 Canoinhas	0.091
430480 Carlos Barbosa	0.091
421490 Rio Fortuna	0.089
411870 Paulo Frontin	0.089
421180 Ouro	0.087
410570 Clevelandia	0.087
421005 Macieira	0.087
355150 Serrana	0.086
420490 Descanso	0.086
312120 Delfinopolis	0.085
410445 Cantagalo	0.085
431805 Sao Domingos do Sul	0.084
312070 Cruzeiro da Fortaleza	0.084
421440 Rio das Antas	0.084
352740 Lucelia	0.083
431440 Pelotas	0.083
311240 Capetinga	0.082
510890 Nova Maringa	0.082
353630 Patrocinio Paulista	0.081
420760 Ipira	0.081
521230 Leopoldo de Bulhoes	0.080
421050 Maravilha	0.079
510622 Nova Mutum	0.079
411820 Paranagua	0.079
420400 Catanduvas	0.079
421940 Witmarsum	0.079
522045 Senador Canedo	0.078
420630 Guabiruba	0.078
520030 Alexania	0.078
420768 Ipuacu	0.078
421190 Palhoca	0.078
421725 Sao Pedro de Alcantara	0.078
312970 Ibiraci	0.078
352930 Matao	0.078
420120 Antonio Carlos	0.078
421890 Urubici	0.077
420970 Lebon Regis	0.077
522119 Terezopolis de Goias	0.076
411860 Paula Freitas	0.076
431290 Nova Bassano	0.076
110030 Vilhena	0.075
420920 Lacerdopolis	0.075
420510 Dona Emma	0.074
520580 Corumba de Goias	0.074
521010 Ipameri	0.074
420315 Calmon	0.074
421210 Palmitos	0.073
351440 Dracena	0.073

421150 Nova Trento	0.073
313030 Iguatama	0.073
421460 Rio do Oeste	0.072
420270 Botuvera	0.072
311980 Corrego Danta	0.071
420540 Florianopolis	0.071
510268 Campos de Julio	0.071
420040 Agua Doce	0.071
421580 Sao Bento do Sul	0.071
420610 Grao Para	0.070
412700 Teixeira Soares	0.070
520815 Gameleira de Goias	0.070
510336 Conquista D'Oeste	0.070
352510 Jardinopolis	0.070
353050 Mococa	0.069
410950 Guaraquecaba	0.069
421507 Riqueza	0.069
420915 Jose Boiteux	0.068
412510 Sao Joao do Triunfo	0.068
420675 Ibiam	0.068
311995 Corrego Fundo	0.068
353360 Nuporanga	0.068
510525 Lucas do Rio Verde	0.068
430320 Cacique Doble	0.068
410965 Honorio Serpa	0.067
421400 Presidente Getulio	0.066
420330 Campo Alegre	0.066
510675 Pontes e Lacerda	0.065
350940 Cajuru	0.065
312125 Delta	0.065
420180 Atalanta	0.064
510830 Uniao do Sul	0.064
431030 Ilopolis	0.064
310190 Alpinopolis	0.064
421660 Sao Jose	0.063
412550 Sao Jose dos Pinhais	0.063
420435 Cordilheira Alta	0.063
421680 Sao Jose do Cerrito	0.063
430210 Bento Goncalves	0.063
420890 Jaragua do Sul	0.063
431730 Santa Vitoria do Palmar	0.062
351480 Eldorado	0.062
432260 Venancio Aires	0.062
352760 Luis Antonio	0.061
354650 Santa Ernestina	0.061
351620 Franca	0.061
352890 Mariapolis	0.061
353640 Pauliceia	0.061
520551 Cocalzinho de Goias	0.061
311430 Carmo do Paranaiba	0.060
420060 aguas Mornas	0.060
510050 Alto Paraguai	0.060
421630 Sao Joao Batista	0.060
412760 Tijucas do Sul	0.060
420550 Fraiburgo	0.060
420590 Gaspar	0.059

431880 Sao Lourenco do Sul	0.059
430450 Cangucu	0.059
520355 Bonfinopolis	0.059
313880 Luz	0.059
420030 Agronomica	0.059
431410 Passo Fundo	0.059
352970 Miguelopolis	0.059
421060 Massaranduba	0.059
410480 Cascavel	0.059
350925 Cajati	0.059
310410 Arceburgo	0.058
316294 Sao Jose da Barra	0.058
312860 Guarda-Mor	0.058
420910 Joinville	0.057
352430 Jaboticabal	0.057
510792 Sorriso	0.057
353310 Nova Guataporanga	0.057
520025 Aguas Lindas de Goias	0.057
421970 Xaxim	0.056
352600 Junqueiropolis	0.056
522060 Silvania	0.056
430790 Farroupilha	0.056
510370 Feliz Natal	0.056
420450 Corupa	0.055
432290 Viadutos	0.055
430165 Barao	0.055
353490 Pacaembu	0.055
431442 Picada Cafe	0.055
430910 Gramado	0.055
420243 Bocaina do Sul	0.055
420080 Anchieta	0.055
410130 Antonio Olinto	0.055
521730 Pirenopolis	0.054
314310 Monte Carmelo	0.054
315640 Romaria	0.054
353160 Monte Castelo	0.054
420470 Cunha Pora	0.054
420360 Campos Novos	0.053
354290 Ribeirao Bonito	0.053
350100 Altinopolis	0.053
421170 Orleans	0.053
355090 Sao Simao	0.053
510590 Nobres	0.052
410960 Guaratuba	0.052
353950 Pitangueiras	0.052
421030 Major Vieira	0.052
430700 Erechim	0.051
354490 Sales Oliveira	0.051
510515 Juina	0.051
354090 Pradopolis	0.050
510455 Itauba	0.050
420110 Anitapolis	0.050
130290 Maues	0.050
431971 Sao Valentim do Sul	0.050
