# Supplementary figures and images for: Spatiotemporal Dynamics of Hantavirus Cardiopulmonary Syndrome Transmission Risk in Brazil
Source: Viruses. 2019 Oct 31;11(11):1008. doi: 10.3390/v11111008 (PMC6893581; doi:10.3390/v11111008)

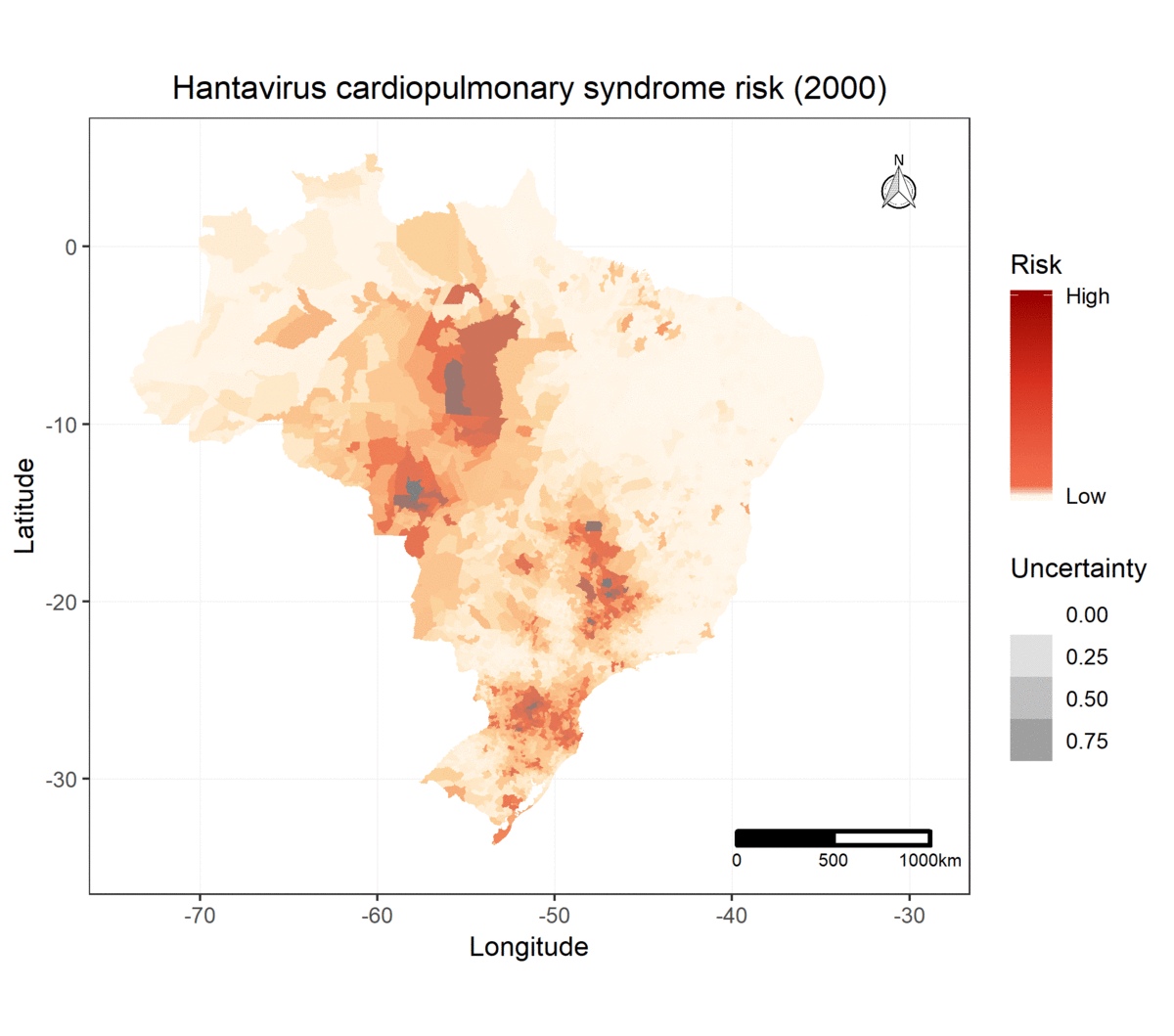

Supplement: Supplementary file 1 [file viruses-11-01008-s001.zip › viruses-631319-for conversion final/for conversion-GIF_S1.gif]
